# ctxR: Utilities for interacting with the CTX APIs

**DOI:** 10.1016/j.namjnl.2025.100031

**Published:** 2025-07-16

**Authors:** Paul M. Kruse, Caroline L. Ring, Katie Paul Friedman, Madison Feshuk, Jason Brown, Carter Thunes, Asif Rashid

**Affiliations:** a United States Environmental Protection Agency, Office of Research and Development, Center for Computational Toxicology and Exposure, 109 T.W. Alexander Drive, P.O. Box 12055, Research Triangle Park, 27709, NC, United States of America; b Oak Ridge Institute for Science and Education, P.O. Box 117, Oak Ridge, 37831-0117, TN, United States of America

**Keywords:** Cheminformatics, Environmental chemistry, Computational toxicology, Computational exposure, Data science, R

## Abstract

A new R package, ctxR, is presented. ctxR is an open-source R Client package used to interact with the Computational Toxicology and Exposure Application Programming Interfaces (CTX APIs) in R. The CTX APIs were developed by the US EPA’s Center for Computational Toxicology and Exposure, and provide users transparent, reproducible access in a programmatic manner to the data surfaced on the CompTox Chemicals Dashboard (CCD) and other data sources. The utility of this package is demonstrated through a case study that compares traditional Points of Departure (PODs) and PODs based on New Approach Methods (NAMs), referred to as PODNAM, relative to estimates of external exposure as an indicator of level of risk. This analysis combines a wide variety of data and showcases the power of programmatic data access that ctxR can provide to the scientific community.

## Highlights

• ctxR is an R package to access US EPA chemical databases via APIs.
• Accessible data include chemistry, hazard, in vitro bioactivity, exposure, and more.
• ctxR provides transparent, reproducible, programmatic access to chemical data.
• ctxR integrates chemical data queries into automated R-based analysis workflows.
• ctxR is demonstrated with a NAM-based chemical prioritization case study.

## 1 Introduction

The CompTox Chemicals Dashboard (CCD), developed by the United States Environmental Protection Agency (US EPA) Center for Computational Toxicology and Exposure (CCTE), is a publicly accessible user interface that integrates data available for chemical substances to facilitate chemical risk assessment and prioritization from multiple domains. Types of data available for chemicals on the CCD may include, but are not limited to, curated chemistry, physicochemical property, environmental fate and transport, exposure, functional use, toxicokinetics, hazard information including *in vivo* toxicity and safety, bioactivity from *in vitro* bioassays, and modeled predicted values. The CCD was first described in Williams et al. (2017), and functionality and data sources included have been continuously expanded since. The CCD has a diverse userbase including scientists and risk assessors both at US EPA as well as across other US and international governmental agencies, private industry, non-governmental organizations, and academia. It has become an indispensable tool for many researchers.

The CCD homepage, https://comptox.epa.gov/dashboard/, offers basic search modes by chemical name or identifier, product use category, and assay/gene name. For a single-substance search, the user can enter a variety of chemical identifiers, including chemical name, common name, Chemical Abstract Service Registry Number (CASRN), InChiKey, or US EPA Distributed Structure-Searchable Toxicity (DSSTox) identifier (DTXSID). Type-ahead search returns a predicted list of possible matches, but exact string and identifier substring search is also possible. The user selects one by clicking on it and is then taken to the Chemical Details page for that substance.

Additional options under the CCD “Search” tab include “Advanced Search” and “Batch Search”. “Search” > “Advanced Search” supports mass spectrometry analyses by allowing the user to identify substances matching a specified mass or a specified molecular formula. The user is presented with a tabular list of matching substances and can either click on each substance to view its CCD Chemical Details page or select substances from the list to send to a batch search.

The CCD homepage also has a “Lists” tab. Selecting “Lists” > “Lists of Chemicals” provides access to curated lists of chemicals maintained by the US EPA. These are curated lists of chemicals grouped by topics such as regulatory status and usage (e.g., antibiotics), and chemicals can be sent to a batch search for downloading additional information of interest.

From “Search” > “Batch Search”, the user either interacts with the chemicals sent to batch search from “Advanced Search” or “Lists of Chemicals”, or enters their list of search inputs, one input per line, into a batch-search box. Only 10,000 inputs are allowed at a time. The user selects the checkbox corresponding to the type(s) of inputs, such as chemical identifiers, monoisotopic masses, or molecular formulas. Then, the user selects “Display All Chemicals” to display the list of substances matching the batch-search inputs, or “Choose Export Options” to choose options for exporting customized results to file download. The export can include data from most of the domains available on an individual substance’s CCD page, as selected by the user. By default, the file is downloaded with a name formatted to include the time and date of download, as “CCD-batch-Search_YYYY_MM_DD_HH_MM_SS”.

Research workflows utilizing the CCD web-based batch search and export may look something like this:

1. The process begins by identifying a list of chemical search terms of interest. These inputs may include chemical names, CASRNs, DTXSIDs, or InChIKeys pulled from a variety of source documents. This original dataset may also include information pertinent for downstream analysis, e.g. environmental monitoring data or new measurements of *in vitro* bioactivity.
2. Search terms of interest are compiled into a spreadsheet. Often, this is done by importing one source dataset into a programming environment, such as R or Python, to do some data wrangling (*e.g.*, to select only the unique substance identifiers, to clean up improperly formatted CASRNs, etc.). Then, the search terms are saved in an Excel, CSV (comma-separated variable), or text file, with one search term per row.
3. Search terms are copied and pasted from the spreadsheet into the CCD Batch Search box. If there are more than 10,000 total search terms, these will need to be divided into batches of 10,000 identifiers or less; then each batch is searched separately.
4. CCD Batch Search Export options are selected to include desired data in the export.
5. CCD Batch Search results are exported and downloaded as another spreadsheet.
6. Steps 3–5 are repeated for each batch of 10,000 identifiers produced in step 2.
7. The resulting spreadsheet(s) of CCD data are imported into R or Python.
8. The CCD datasets are merged with the original dataset.
9. Once this initial data retrieval and integration is complete, data analysis and visualization can begin.

This workflow includes several manual steps such as copying and pasting the search terms into the CCD Batch Search page, selecting export options, downloading the resulting spreadsheets, and importing the downloaded into the computational analysis environment. At any of these stages, human error can occur. Here are a few real-world possibilities:

• Researchers can unintentionally copy and paste the wrong search terms into the CCD Batch Search, especially if they have more than 10,000 identifiers and must allocate into them into separate batches for searching.
• If a researcher opens a CSV file using Microsoft Excel, any values that appear to be in date-like format may be converted to a date (depending on Excel version). For example, a hypothetical CASRN of “2017-11-4” may automatically be converted to an MM/DD/YYYY-formatted date of “11/04/2017”.
• Researchers could accidentally rename a downloaded CCD data file to overwrite a previous download.
• Researchers could mistakenly import the wrong CCD download file into R to merge with their data of interest.

Considered somewhat tedious and error-prone, the manual stages of this workflow are neither transparent nor easily reproducible because options for this process are typically not fully documented by users. Further, the CCD version and the versions of the underlying data may not be noted by users. In any case, interacting with previous versions of CCD is not currently enabled.

Some of these manual batch-search steps could be avoided if users could access the full databases underlying the CCD, so that programmatic queries could be constructed. In fact, users can download or otherwise access full copies of much of the data underlying the CCD, such as the Toxicity Forecaster (ToxCast) database invitrodb and the data contained in the high-throughput toxicokinetics R package httk. However, interacting with these data requires learning the structure of diverse data objects and wrangling data from multiple heterogeneous sources.

As a machine-based, reproducible alternative to the web-based batch search, CCTE has introduced a public Application Programming Interface (API) to allow programmatic access to the CCD and other computational toxicology and exposure (CTX) resources, aptly named the “CTX APIs”. The CTX APIs allow researchers to automate data searches through functionalized queries, removing the need for manual data import or export steps and laborious data wrangling from databases with diverse data structures.

However, CCD users wishing to access data through the CTX APIs may be new to using an API and unsure where to start. Although researchers may be familiar with writing code in software languages such as R, they may be unfamiliar with how to construct API calls or parse the data returned. To make this process easier, a client package has been developed to make interacting with the CTX APIs as easy as writing a single line of R code. This is ctxR.

The implementation and usage of ctxR will be described. Then, a case study will be described that demonstrates the utility of ctxR, leveraging a variety of CTX API data across the four data domains to perform a New Approach Methods (NAM)-based analysis of potential chemical hazard and exposure.

## 2 Methods

### 2.1 Implementation

ctxR is implemented as a free, open-source, publicly available R package. ctxR functions are organized according to the CTX APIs resources listed at https://www.epa.gov/comptox-tools/computational-toxicology-and-exposure-apis. There is one ctxR function corresponding to each API resource. They are named accordingly: for example, get_chemical_details() corresponds to the ‘Chemical Details Resource’. Within each resource, there are endpoints facilitating different type of data requests.

The functions of ctxR depend upon functions from the R package httr (Wickham, 2023) to construct API calls, and functions from the R package jsonlite (Ooms, 2014) to parse the resulting JSON data returned in the API response. To interact with the CTX APIs, requests are constructed to include an authentication key and are tailored to each endpoint with input information specific to the endpoint. ctxR leverages the R package httr to construct Hypertext Transfer Protocol (HTTP) requests to send to the API server for information. When a response to the request is received, ctxR again uses the httr package to translate the received HTTP information to more basic R objects. The package jsonlite is used in this process to handle response data and return R objects like base R data.frames or data.tables, depending on the ctxR function the user called. This ensures that the user can interact with the API endpoints using familiar function paradigms and R objects rather than having to learn how to construct and process HTTP requests using httr and jsonlite.

Aside from a handful of functions returning MOL files or molecular image files, most functions in ctxR return data objects or named lists of objects of class data.table (Barrett et al., 2025). data.tables are enhanced data.frames optimized for large scale data manipulation. This standardized object class is used to provide consistency across the different CTX API endpoints and make adoption of the corresponding functions into R workflows as seamless as possible.

APIs may require user authentication to protect data that is being shared or limit traffic to the API infrastructure. For the CTX APIs, users can request a free API key for access. The CTX API key can be stored locally during a single R session or more permanently across all R sessions. For key storage that is persistent across sessions, the credential is written to the user computer and can be replaced at any time. For single session storage, the user executes the function call ctxR::register_ctx_api_key(key = <YOUR_API_KEY>, write = FALSE) (substituting their own API key). For persistent storage, the user executes the same call, but with the parameter write = TRUE instead. ctxR functions automatically access the stored key using ctxR::ctx_api_key() if not supplied with one during the function call, so the user is not burdened with supplying the API key every time they call a function that communicates with the API endpoints. Furthermore, if the user needs to retrieve the key themselves, this can be done using the function ctxR::ctx_api_key().

There are instances in which users may wish to search for data corresponding to several chemicals or assays, for which repeated single item searches would be burdensome. The CTX APIs support batch searching for some endpoints, and ctxR includes functions corresponding to these endpoints. In addition, ctxR extends batch searching to those functions for which the CTX APIs do not provide explicit batch searching endpoints. For those endpoints that support batch searching on the API side, different HTTP methods are used than in single entry endpoints (GET for single entry and POST for batch searching), and these optimized batch searching endpoints provide fast and efficient access to the bulk data downloads. For those endpoints that are not yet optimized for batch searching on the API server side, ctxR uses looping to submit a series of requests for each value included in the input parameters and return response data. There is a built-in option to limit the rate at which these requests are sent by the argument rate_limit in all ctxR functions that query the APIs. The argument rate_limit gives an amount of time to wait between requests, in units of seconds. The default value is currently zero (i.e., no rate limiting), but as user traffic increases, more robust rate limiting may be implemented within the package to protect infrastructure and maintain API performance for all users. However, even with rate limiting in place, the CTX APIs return data quickly; any limit should impose a minimal impact for most users. For instance, using get_chemical_details_batch() to query 500 chemicals returns in 1.75 s without any rate limiting, but the same request with a value of 0.5 s for the rate_limit parameter returns a response in 4.09 s. For most CTX APIs users, this discrepancy does not appreciably impact the overall time spent retrieving data.

### 2.2 Usage

Any use of the CTX APIs – including through ctxR – requires that the user be assigned a unique API key. API keys are free and publicly available, but they must be requested. To request an API key, contact ccte˙api@epa.gov. ctxR can be downloaded from both CRAN and the public USEPA GitHub page. To install the latest stable ctxR version from CRAN (version 1.1.2 at the time of this publication), use install.packages(‘ctxR’). To install the latest development version from GitHub (version 1.1.0.9000 at the time of this publication), use devtools::install_gitub(‘USEPA/ctxR’). Once the package is installed, the user can access an introductory vignette that leads the user through setting up the API key for access in a single R session or for persistent access across R sessions. This vignette can be accessed using the R command vignette(topic = ‘Introduction’, package = ‘ctxR’).

The CTX APIs are organized into four data domains: Chemical, Bioactivity, Hazard, and Exposure. Each domain has several API endpoints that provide access to a variety of different types of data. For instance, the CTX Chemical API contains endpoints that make available various physico-chemical property data, chemical lists, molecular structure files, and image data, as well as provide chemical identifier search functionality. A few of these endpoints could be combined to provide a user with the ability to search for a chemical by a name, retrieve physico-chemical property data, and retrieve structures of that chemical in a variety of formats. This functionality can prove useful when designing workflows that assimilate a wide variety of data for modeling purposes. ctxR includes vignettes that provide an overview and examples of functions for each data domain (vignettes ‘Bioactivity’, ‘Chemical’, ‘Exposure’, and ‘Hazard’). Each data domain vignette provides code examples on how to use different functions, and supplies references for papers and additional resources associated with that data domain. API data domain documentation can be found at https://www.epa.gov/comptox-tools/computational-toxicology-and-exposure-apis-data-domains. See Tables 1, 2, 3, and 4 for a detailed description of the available endpoints and functions.

The CTX Chemical API includes data from the Distributed Structure-Searchable Toxicity (DSSTox) database (USEPA, 2024), predictions from the Toxicity Estimation Software Tool (TEST) Quantitative Structure Activity Relation (QSAR) models (Martin, 2020), predictions from the Open (Quantitative) Structure–activity/property Relationship App (OPERA) (Mansouri et al., 2018), as well as other data published on the CCD.

**Table 1:** Overview of chemical data domain including chemical resources, endpoints, and the associated ctxR functions.

CTX API Resource	Endpoint	ctxR function
	POST: Get data by the batch of dtxsids	get_chemical_details_batch()
Chemical Details	POST: Get data by the batch of dtxcids	get_chemical_details_batch()
	GET: Get data by the batch of dtxsids	get_chemical_details()
	GET: Get data by the batch of dtxcids	get_chemical_details()

Pubchem Link to	POST: Check existence for batch of dtxsids	checl_existence_by_dtxsid_batch()
GHS Classification	GET: Check existence by dtxsid	check_existence_by_dtxsid()

	GET: Get chemical properties by id and range	get_chemical_by_property_range()
Chemical Property	GET: Get properties by dtxsid	get_chem_info()
	GET: Get property id by type=predicted	get_chem_info(type=‘predicted’)
	GET: Get property ids by type=experimental	get_chem_info(type=‘experimental’)

Chemical Fate	POST: Get data by the batch of dtxsids	get_fate_by_dtxsid_batch()
	GET: Get data by dtxsid	get_data_by_dtxsid()

	POST: Search by exact batch of values	chemical_equal_batch()
	POST: Search ms ready chemical by batch of mass range	get_ms_ready_by_mass_with_error_batch()
	GET: Search by starting value	chemical_starts_with()
Chemical Search	GET: Search by exact value	chemical_equal()
	GET: Search by substring value	chemical_contains()
	GET: Search ms ready chemical using mass range	get_msready_by_mass()
	GET: Search ms ready chemicals by formula	get_msready_by_formula()
	GET: Search ms ready chemicals by DTXCID	get_msready_by_dtxcid()

	GET: Get all list types	
	GET: Get public lists by type	get_chemical_lists_by_type()
	GET: Get public list by name	get_public_chemical_list_by_name()
	GET: Get list names by dtxsid	get_lists_containing_chemical()
Chemical List	GET: Get dtxsids for starting value	get_chemicals_in_list_start()
	GET: Get dtxsids for exact value	get_chemicals_in_list_exact()
	GET: Get dtxsid for contain value	get_chemicals_in_list_contain()
	GET: Get dtxsids for list name	get_chemicals_in_list()
	GET: Get all public lists	get_all_public_chemical_list()

	GET: Get mrv file by dtxsid	get_chemical_mrv()
	GET: Get mrv file by dtcsid	get_chemical_mrv()
	GET: Get mol file by dtxsid	get_chemical_mol()
Chemical File	GET: Get mol file by dtcsid	get_chemical_mol()
	GET: Get structure image by gsid	
	GET: Get structure image by dtxsid	get_chemical_image()
	GET: Get structure image by dtxcid	get_chemical_image()
	GET: Get generated structure image for smiles	get_chemical_image()

Chemical Synonym	POST: Get synonyms by batch of dtxsids	get_chemical_synonym_batch()
	GET: Get synonym by dtxsid	get_chemical_synonym()

**Table 2:** Overview of bioactivity data domain including bioactivity resources, endpoints, and the associated ctxR functions.

CTX API Resource	Endpoint	ctxR function
Bioactivity Assay	GET: Get annotation by aeid	get_annotation_by_aeid()
	GET: Get all assays	get_all_assays()

	GET: Get summary by aeid	get_bioactivity_summary()
	GET: Get data by spid	get_bioactivity_details()
Bioactivity Data	GET: Get data by m4id	get_bioactivity_details()
	GET: Get data by dtxsid	get_bioactivity_details()
	GET: Get data by aeid	get_bioactivity_details()

**Table 3:** Overview of hazard data domain including hazard resources, endpoints, and the associated ctxR functions.

CTX API Resource	Endpoint	ctxR function
	POST: Get all data by batch of dtxsids	get_hazard_by_dtxsid_batch()
	POST: Get human data by batch of dtxsids	get_human_hazard_by_dtxsid_batch()
	POST: Get eco data by batch of dtxsids	get_ecotox_hazard_by_dtxsid_batch()
	GET: Get all data by dtxsid	get_hazard_by_dtxsid()
	GET: Get human data by dtxsid	get_human_hazard_by_dtxsid()
	GET: Get eco data by dtxsid	get_ecotox_hazard_by_dtxsid()

Skin Eye	POST: Get data by batch of dtxsid	get_skin_eye_hazard_batch()
	GET: Get data by dtxsid	get_skin_eye_hazard()

Cancer Summary	POST: Get data by batch of dtxsids	get_cancer_hazard_batch()
	GET: Get data by dtxsid	get_cancer_hazard_batch()

	POST: Get summary data by batch of dtxsids	get_genetox_summary_batch()
	POST: Get details data by batch of dtxsids	get_genetox_details_batch()
Genetox	GET: Get summary data by dtxsid	get_genetox_summary()
	GET: Get details data by dtxsid	get_genetox_details()

**Table 4:** Overview of exposure data domain including exposure resources, endpoints, and the associated ctxR functions.

CTX API Resource	Endpoint	ctxR function
	GET: Find functional use data by dtxsid	get_exposure_functional_use()
Functional Use	GET: Functional use probability by dtxsid	get_exposure_functional_use_probability()
	GET: Exposure functional use category	get_exposure_functional_use_category()

Product Data	GET: Find product data by dtxsid	get_exposure_product_data()
	GET: Exposure product data PUC	get_exposure_product_data_puc()

httk Data	GET: Find httk data by dtxsid	get_httk_data()

List Presence	GET: Exposure list presence tags	get_exposure_list_presence_tags()
	GET: Find list presence data by dtxsid	get_exposure_list_presence_tags_by_dtxsid()

General Exposure	GET: Find general exposure prediction data by dtxsid	get_general_exposure_prediction()
Prediction		

Demographic Exposure	GET: Exposure SEEM demographic by dtxsid	get_demographic_exposure_prediction()
Prediction		

The CTX Bioactivity API contains data sourced from the ToxCast database, invitrodb (USEPA, 2023b). This publicly available resource of targeted bioactivity assay screening data for thousands of chemicals and is managed by the ToxCast pipeline R package tcpl (Feshuk et al., 2023). In fact, the latest release of tcpl, version 3.2.0, has integrated with ctxR for accessing data made available through the CTX Bioactivity API endpoints. Regular ToxCast users may find it easier to use tcpl, which has integrated ctxR’s functions to make API data retrievable in a familiar format. However, for workflows incorporating various data domains, ctxR provides the broad access a user may desire.

The CTX Hazard API consists of several endpoints that surface data from the US EPA Toxicity Values database, ToxValDB (Judson, 2022). ToxValDB is a large compilation of human health-relevant *in vivo* toxicology data, including data on both *in vivo* toxicity experiments and derived toxicity and guideline values aggregated from over 40 sources, including government agencies, both at the state and federal level, domestic and foreign. ToxValDB was designed to provide high-level summary data in a standardized format to facilitate comparison and data use across many individual databases. The database includes hundreds of thousands of data records on hazard, skin-eye irritation, cancer, and genotoxicity for thousands of chemicals.

The CTX Exposure API encompasses exposure data, both curated and predicted, from several data different sources. In addition to the CCD, the Chemical Exposure Knowledgebase (ChemExpo) (USEPA, 2023a) was developed to provide access to data stored in the Consumer Products Database (CPDat) (Dionisio et al., 2018), which contains information on thousands of chemicals that are used within commerce.

The data available through ChemExpo is organized into three main categories: functional use data, product use data, and list presence data. Functional use data describes the documented and predicted functional uses for a given chemical (e.g., ‘Pharmaceutical (EPA)’, ‘Flavouring and nutrient’, ‘Softener and conditioner’, ‘Biocide’, ‘Fragrance’, ‘Deodorizer’ for Caffeine). Product use data describes the different types of products for which a given chemical is an ingredient and the broad product use categories that contain the chemical. These product use categories (PUCs) are organized hierarchically (e.g., the product type ‘hand/body lotion’ contained in the product family ‘general moisturizing’ contained in the general category ‘Personal care’ for one product listing associated to Caffeine). List presence data include keywords that describe the type of uses associated to a chemical (e.g., ‘pharmaceutical’ for Caffeine). These keywords are also used to tag and identify documents of interest found within ChemExpo that contain information on the chemical.

Exposure data predictions are also available from the SEEM models (SEEM3 among them) and the R package httk (Ring et al., 2018, Pearce et al., 2017). The httk data includes predicted values under different models for various toxicokinetic parameters. These are relevant to users performing *in vitro* to *in vivo* extrapolation (IVIVE) and toxicokinetic research. The SEEM models provide data on predicted exposure pathways for general population and specific concentration values for populations broken down by demographics. There are additional models beyond the SEEM predictions included as well, such as SHEDS-HT, RAIDAR, and several USETOX models among others (Isaacs et al., 2014, Arnot et al., 2006, Rosenbaum et al., 2011).

### 2.3 Case study

To demonstrate the utility of ctxR, a case study was conducted that leveraged a variety of CTX API data across the four data domains. At the time of publication, data versions for the domains are as follows: “20240208_DSSTox” for DSSTox, “20191118_ChemPROP” for PhysChem and Environmental Fate/Transport Properties, v2.6 for OPERA, invitrodb v4.1 for ToxCast Bioacivity Data, CPDat 4.0.0-alpha.3 for Product & Use Categories, EPA QSURs, Chemical Weight Fraction, and Collected Data on Functional Use, Predicted Probability of Associated Functional Use via the R package QSUR v0.0.0.9000, and ToxValDB v9.2 for Toxicity Values.

Determining whether a chemical may pose a risk to human health has traditionally relied on collecting data from *in vivo* studies using different dosing regimens and study designs of the target chemical within various animal models. More recently, there has been an effort to move away from animal testing and instead focus on New Approach Methods (NAMs), combining in silico modeling with high-throughput assay screening data. As more NAMs are developed for addressing data gaps in chemical assessments, one component of building scientific confidence in use of NAMs for data-poor chemicals is characterization of whether NAM-based methods produce information that is protective for observations from traditional toxicity testing methods.

Points of departure (PODs) are quantitative values that define the dose associated with a bioactivity or hazard of interest. In Paul Friedman et al. (2020), the authors conducted a study comparing the results of NAM-derived PODs predictions to *in vivo*-based POD predictions. Specifically, PODs derived from curated hazard data, representing traditional PODs, were compared to PODs derived from NAMs using bioactivity estimates from ToxCast combined with toxicokinetic modeling via the Rpackage, httk. When these traditional PODs and NAM-derived PODs were compared, NAM-derived PODs were in general more conservative based on the options applied in the case study. The chemicals for which traditional PODs were more conservative were further examined to determine why the NAM predictions were less protective relative to the other chemicals. A Bioactivity:Exposure Ratio (BER) was also examined to compare the PODs with exposure predictions from the SEEM2 model (Wambaugh et al., 2014) as a means of demonstrating prioritization for further information gathering.

The analysis of Paul Friedman et al. (2020) was conducted by wrangling several large, disparate data sets representing *in vivo* toxicity data, *in vitro* bioactivity data, toxicokinetic data, and exposure data. Now, with ctxR, data acquisition and integration for a similar analysis is greatly simplified.

The analysis of Paul Friedman et al. (2020) was replicated using ctxR functionality by obtaining and integrating data for a similar analysis using updated data sources available via the CTX APIs. To this end, chemicals from the list of all chemicals that have httk simulation data were first obtained. The function get_ chemicals_in_list() was used to retrieve the curated list of chemicals included in the ‘HTTKHUMANv2’ (as of version httk v2.5.0) chemical list, also available on the CCD (USEPA, 2025). Returned data consisted of chemical identifiers, molecular mass, and literature-related metrics among other types. Any associated exposure data for these chemicals was then retrieved using the function get_demographic_ exposure_prediction_batch() with an input of the list of DTXSID chemical identifiers from the ‘HTTKHUMANv2’ chemical list. This returned data.frame, which included many different exposure predictors and exposure predictions, was subset to retain only the SEEM3 exposure predictions. Additionally, the httk data for these chemicals were retrieved using the same list of chemical identifier DTXSIDs as an input to the function get_httk_data_batch(). These data included the steady-state plasma concentration ($C_{ss}$) values for a long-term daily dose of 1 mg/kg/day, generated from httk simulations running the 3-compartment steady-state model.

Next, ToxCast bioactivity data was acquired to compute the PODs derived from NAMs for each chemical. The function get_bioactivity_details_batch() was used to retrieve multiple-concentration response data. The returned data was then subset to only retain $AC_{50}$ values for assays where the sample tested was considered active and the curve did not have many cautionary flags. Specifically, only assay data were included with an active hit call (hitc) value greater than or equal to 0.9 and fewer than four cautionary flags indicative of aberrant curve fitting behavior. A 5th percentile $AC_{50}$ was then computed for each chemical. Since $AC_{50}$ values were reported in ‘μmol/L’ and computation of administered equivalent dose using $C_{ss}$ values requires units of ‘mg/L’, the molar mass data for each chemical was used to stoichiometrically convert $C_{ss}$ to μmol/L. With the correct units, $AC_{50}$ in μmol/L divided by $C_{ss}$ in μmol/L for 1 mg/kg/day dosing yields a human administered equivalent dose in mg/kg/day. Both the 50th and 95th percentile estimates of $C_{ss}$ values from the httk three-compartment model, based on variation in $C_{ss}$ in the human population, resulting in two different POD NAM values for each chemical.

Next, POD traditional values were determined by accessing the *in vivo* hazard data via the CTX Hazard API. In this study, only human hazard *in vivo* data accessed via the function get_human_hazard_by_ dtxsid_batch() was considered. As in the analysis completed by the authors in Paul Friedman et al. (2020), hazard-related effect level values from animal studies included No/Lowest Observed Adversed Effect Level (‘NOAEL’/‘LOAEL’) or No/Lowest Observed Effect Level (‘NOEL’/‘LOEL’) values from ToxValDB only if they met certain criteria: only mammalian models relevant to human health; only records in mg/kg-day or units that could be converted to those units, such as in mass/kg-day or molar/kg-day or parts per million (ppm); and only studies that reflected repeat dose animal study designs. As with the $AC_{50}$ values, a 5th percentile of the included effect level value for each chemical was determined as the POD traditional.

**Fig. 1:**
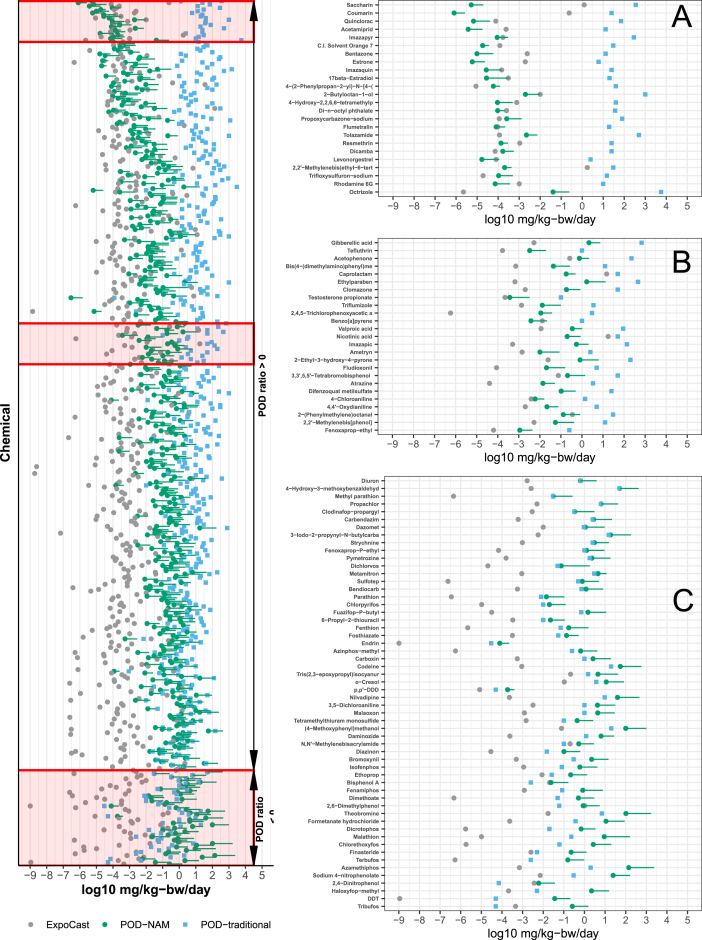
Comparison of PODs derived from NAMs, *in vivo* data, and $C_{ss}$ predictions using 50th and 95th percentile values from httk and exposure predictions from the SEEM3 model. The POD-NAM values calculated from 50th and 95th percentile values of $C_{ss}$ correspond to the left and right ends of the green bars, respectively.

For this case study, SEEM3 exposure predictions were used in the comparison with the POD traditional and POD NAM values, while the previous study examined SEEM2 exposure predictions. To continue the analogous analysis, the log10 values of the SEEM3 exposures, the log10 values of the POD traditional values, and the log10 values of the POD NAM values were examined. POD ratios (calculated as log10 POD traditional – log10 POD NAM) as well as Bioactivity:Exposure Ratios (BERs) were computed using the log10 POD NAM values corresponding to the 95th percentile $C_{ss}$ values (pod.nam.95) and the SEEM3 exposure predictions corresponding to the 95th upper limit (u95). The log10 BER values (ber.95) were calculated as (log10 POD NAM – log10 95th percentile exposure). When log10 BER values are positive, this indicates exposure predictions are less than the log10 POD NAM values, while negative values indicate potential for exposures that exceed NAM-based PODs. See Fig. 1 for a visual representation of the NAM-based PODs, traditional PODs, and SEEM3 exposure predictions.

## 3 Results

Of the 507 chemicals with complete data, 87 had log10 BER values of less than 0, indicating the potential for exposure that could exceed the NAM-based POD for these chemicals. Of these 87, 37 had BERs calculated from 95th percentile exposure and 50th percentile $C_{ss}$ (log10 BER50) values that were greater than 0, indicating that these chemicals may be of less interest than the 50 chemicals that had (log10 BER50) values less than 0.

For those chemicals with log10 BER < 0 but log10 BER50 > 0, the log10 BER values ranged from −1.040 to −0.174 log10 units and the log10 BER50 values ranged between 0.008 and 0.935 log10 units. The BER values from both sets of NAM-derived PODs are nearly all within 1 order of magnitude of unity, so exposure is ten times to one tenth the value of the PODs for these chemicals. For the chemicals with log10 BER < 0 and log10 BER50 < 0, the log10 BER values range between −5.476 and −0.385 while the log10 BER50 values fall between −4.957 and −0.015 log10 units, so both span about five orders of magnitude.

In Paul Friedman et al. (2020), 48 chemicals were identified as having a more conservative traditional POD value than the NAM-derived POD value. Considering that this earlier analysis used different or earlier versions of data from TxoValDB, invitrodb and httk, compared to what is available now on CTX APIs, these chemicals were re-examined these chemicals. Forty-four of 48 chemicals were found to have sufficient data to include in the current analysis. One (‘DTXSID3032464’) was missing from the HTTKHUMANv2 list of chemicals with httk data despite having sufficient httk data from an older version of httk, and three (‘DTXSID6024177’, ‘DTXSID7020508’, and ‘DTXSID3020679’) were missing httk data despite being included in the HTTKHUMANv2 list of chemicals. Of these 44 chemicals with sufficient data, 18 had a more conservative NAM-derived POD, with log10 POD NAM < log10 POD traditional, while 26 had a more conservative traditional POD value than a NAM-derived value. The values of the POD ratios range between −3.713 to 3.657 log10 units, with a median value of −0.08068 log10 units. Of those 26 chemicals with a more conservative traditional POD than a NAM-derived POD, only one had a NAM-derived BER that was negative, (for DTXSID9024063).

Traditional PODs were further explored to examine when instances where the BER using traditional POD was negative while the BER using a NAM-derived POD was positive. Since log10 BER = log10 POD NAM – log10 95th percentile exposure and POD ratio = log10 POD traditional – log10 POD NAM, adding these values yields log10 POD traditional – log10 95th percentile exposure, the BER using the traditional POD and the 95th percentile SEEM3 exposure (log10 BERtrad). This traditional POD BER was negative for 10 chemicals, of which six had NAM-derived BER values that were negative. This means that only four chemicals had a negative traditional POD BER value and positive NAM-derived POD value. Of these four, three had NAM-derived BER values between 0.063 and 0.420 log10 units (‘DTXSID7020182’, ‘DTXSID0020523’, ‘DTXSID8025595’), while the fourth had a value of 2.753. These results imply that using the NAM-derived POD provides a relatively good value for protective BER values, and even when this POD is not as conservative as the traditional POD, the NAM-derived BER values are still generally protective.

Comparing the NAM-derived POD values with the traditional POD values, 452 chemicals had more conservative log10 POD NAM than log10 POD traditional, while 55 had more conservative log10 POD traditional than log10 POD NAM. When using 50th percentile POD (pod.nam.50), 401 chemicals had more conservative NAM derived POD values than traditional values, while 106 had more conservative traditional POD values than NAM-derived POD values. See Table 5 for a breakdown of these numbers.

**Fig. 2:**
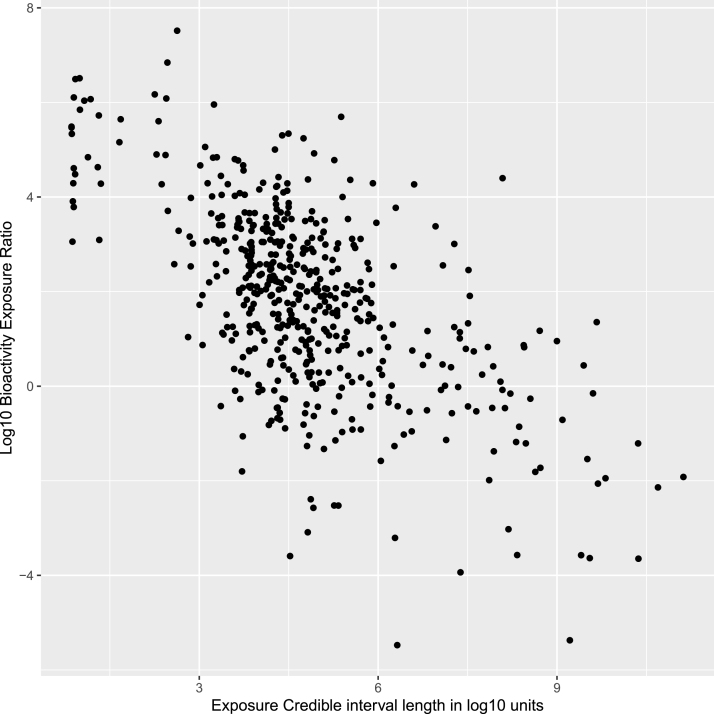
Comparison of interval length of upper and lower 95th percentile exposure values with log10 BER values.

There is a correlation of −0.617 between the log10 size of the 95th percentile credible interval for SEEM3 exposures (the log10 difference of the upper 95th percentile and lower 95th percentile values log10(u95) – log10(l95)) and the BER using NAM-derived POD and 95th percentile exposure value, log10 BER. This suggests that the more certain the exposure estimate (shorter log10 length of 95th credible interval of exposure), the higher the BER, while the more uncertain the exposure value, the lower the BER. See Fig. 2 for a plot of these values.

**Table 5:** Comparison of POD NAM and POD traditional using 50th and 95th percentile $C_{ss}$ values.

	More conservative	
	POD NAM	POD traditional
50th percentile $C_{ss}$	452	55
95th percentile $C_{ss}$	401	106

## 4 Discussion

The ctxR R package provides an extension of the US EPA’s publicly released computational toxicology and exposure resources via the CTX APIs. These data resources are often provided in multiple formats, including database downloads or interactive graphical user interfaces like the CompTox Chemicals Dahsboard. APIs provide an alternative to connect this data that would otherwise require careful documentation of manual access processes that are often prone to human error. CTX APIs are a centralized location for the collection of these data resources available for discovery and reuse by researchers and tool developers. ctxR acts as an API R Client to simplify the HTTP interaction in a reproducible manner.

A case study has been presented that demonstrates the utility of integrating ctxR into NAM-based computational toxicology workflows. The authors in Paul Friedman et al. (2020) completed a detailed analysis of potency estimates derived from traditional animal toxicity testing compared to alternatives and exposure levels. Their work entailed combining several data sources, including Toxcast *in vitro* bioactivity data and *in vivo* hazard data from the ToxValDB, and running toxicokinetic and exposure simulations. Using ctxR, a similar analysis was demonstrated by accessing all data (physico-chemical properties, hazard, bioactivity, httk, and exposure data) through API calls in roughly 25 lines of code compared to the original analysis, which included roughly 1000 lines of code to retrieve the data, compile the data, and standardize the data for integration. A similar data wrangling and filter procedure was used to prepare the data for analysis.

One main advantage to employing ctxR in this workflow is the increased automation. It is preferred to rerun the workflow against the updated data version on the CTX APIs over the alternative of downloading new flat files or configuring a new database version. The user should ensure data quality control is consistent between different runs of the workflow with updated data, though this requires less effort than constructing the data preparation and analysis from scratch when new data are released. Hence, while not nearly as detailed as in Paul Friedman et al. (2020), this less complex analysis can be run with a handful of keystrokes and has the potential to provide a broad overview of the state of NAM-derived PODs relative to traditional PODs. As more *in vivo*, *in vitro*, and httk data become available, researchers can check how well NAMs are providing protective estimates of chemical hazard and exposure in comparison to traditional risk assessment efforts in a scheduled and fully automated fashion.

Moreover, the case study illustrates how manually curated analyses that use data available on the CTX APIs may also be automated and run periodically. This can allow researchers to run such analyses in a screening-level approach and pinpoint those chemicals that warrant further attention in a more human-intensive effort. Additionally, such automated approaches can elucidate data gaps by identifying chemicals missing data at various steps, which in turn may prompt future research efforts.

## 5 Conclusion

The R package ctxR presents a programmatic, transparent, reproducible approach to accessing computational toxicology and exposure data resources developed by the US EPA. This software package streamline the R workflows that researchers use to source and wrangle data needed for various projects. ctxR has been successfully utilized in internal workflows and early collaborations. Robust download numbers comparable to other well-known and oft-used US EPA R packages suggest widespread adoption of this software from its initial release, confirming the need for this software to ensure transparent, reproducible data workflows.ctxR will be iteratively updated to align with CTX APIs updates and address the continued data needs for next generation risk assessment and regulatory decision-making applications.

The US EPA is a world leader in releasing high quality, expert-driven computational toxicology and exposure resources for public consumption. The CTX APIs are a relatively new platform for distributing data with growing interest from internal stakeholders and external partners. As user feedback and needs continue to shape the data and format of data presented, continued growth and development of the CTX APIs will enable seamless data integration, foster more collaboration, and promote new research opportunities via data accessibility. Consequently, development of additional functions to cover endpoint updates as well as new endpoints will ensure the ctxR also has a robust future of development and use in the coming years. Furthermore, versioning of both the CTX APIs and data accessed will be featured in the future, ensuring greater reproducibility for those seeking to replicate the results of workflows and corresponding analyses.

Moreover, with CTX API client packages being developed in other languages (notably Python and Java), ctxR will be one package within a CTX API client package suite supporting a community of developers looking to harmonize access and data standards across several computational platforms and coding environments. This coordinated effort will ensure that ctxR meets open-source standards enforced by CRAN as well as development and design standards set forth by other API clients being developed and maintained by US EPA researchers.

In conclusion, the ctxR package is an important tool to enable the use of NAM-based toxicology and exposure data alongside traditional data, and to enable the ongoing development of computational NAMs that integrate data science, statistics, and machine learning approaches to answer critical questions about chemical safety.

## Disclaimer

The views expressed in this publication are those of the authors and do not necessarily represent the views for policies of the U.S. Environmental Protection Agency. Reference to commercial products or services does not constitute endorsement.

## CRediT authorship contribution statement

**Paul M. Kruse:** Writing – review & editing, Writing – original draft, Visualization, Validation, Software, Methodology, Investigation, Formal analysis, Conceptualization. **Caroline L. Ring:** Writing – review & editing, Writing – original draft, Supervision, Project administration, Methodology, Conceptualization. **Katie Paul Friedman:** Writing – review & editing, Visualization, Validation, Methodology, Conceptualization. **Madison Feshuk:** Writing – review & editing, Software, Methodology, Data curation. **Jason Brown:** Writing – review & editing, Software, Resources, Project administration, Data curation. **Carter Thunes:** Writing – review & editing, Software, Methodology, Data curation. **Asif Rashid:** Writing – review & editing, Supervision, Software, Resources, Project administration, Methodology, Data curation.

## Declaration of competing interest

The authors declare that they have no known competing financial interests or personal relationships that could have appeared to influence the work reported in this paper.

## Data Availability

As part of EPA’s commitment to provide “open data”, these CTX APIs are publicly available data resources for anyone to access and use ( https://www.epa.gov/comptox-tools/computational-toxicology-and-exposure-apis). These APIs are hosted on cloud.gov, a secure cloud environment managed by the General Services Administration specifically for U.S. federal government applications. These data are free of all copyright restrictions, and are fully and freely available for both non-commercial and commercial use. All data are also available for download on the Downloadable Computational Toxicology Data page ( https://www.epa.gov/chemical-research/downloadable-computational-toxicology-data). The results in this paper were obtained using R version 4.4.1 with the ctxR package version 1.1.1. ctxR is available at https://CRAN.R-project.org/package=ctxR. R itself and all other packages used are available from the Comprehensive R Archive Network (CRAN) at https://CRAN.R-project.org/.

## Appendix A Supplementary data

MMC S1 .
